# Precision Interventional Brachytherapy: A Promising Strategy Toward Treatment of Malignant Tumors

**DOI:** 10.3389/fonc.2021.753286

**Published:** 2021-10-08

**Authors:** Pan He, Siwen Guan, En Ren, Hongwei Chen, Hu Chen, Yisheng Peng, Bin Luo, Yongfu Xiong, Bo Li, Jingdong Li, Jingsong Mao, Gang Liu

**Affiliations:** ^1^ State Key Laboratory of Molecular Vaccinology and Molecular Diagnostics, Center for Molecular Imaging and Translational Medicine, School of Public Health, Xiamen University, Xiamen, China; ^2^ Department of General Surgery (Hepatobiliary Surgery), The Affiliated Hospital of Southwest Medical University, Luzhou, China; ^3^ Institute of Hepato-Biliary-Intestinal Disease, Department of Hepatobiliary Surgery, Affiliated Hospital of North Sichuan Medical College, Nanchong, China; ^4^ Department of Radiology, Xiang’an Hospital of Xiamen University, Xiamen, China

**Keywords:** precision brachytherapy, interventional surgery, radiopharmaceuticals, drug delivery, tumor treatment

## Abstract

Precision interventional brachytherapy is a radiotherapy technique that combines radiation therapy medicine with computer network technology, physics, *etc.* It can solve the limitations of conventional brachytherapy. Radioactive drugs and their carriers change with each passing day, and major research institutions and enterprises worldwide have conducted extensive research on them. In addition, the capabilities of interventional robotic systems are also rapidly developing to meet clinical needs for the precise delivery of radiopharmaceuticals in interventional radiotherapy. This study reviews the main radiopharmaceuticals, drug carriers, dispensing and fixation technologies, and interventional robotic precision delivery systems used in precision brachytherapy of malignant tumors. We then discuss the current needs in the field and future development prospects in high-precision interventional brachytherapy.

## Introduction

Malignant tumors (MT) are among the most severe diseases threatening human health conditions in the 21st century. MT are also the focus of substantial research worldwide (1–3). The primary treatment modalities for MT include surgical treatment, radiotherapy, chemotherapy, immunotherapy, as well as the newly developed photothermal, photodynamic, and sound-dynamic therapies (4–7). Of these, comprehensive surgical treatment mainly based on surgical treatment plays a crucial role in the treatment of MT (8). However, it is often difficult, if not impossible, to diagnose MT early. Accordingly, by the time tumors are found, patients are in the middle and late stage of the disease, and the rate of surgical resection and radical cure is thus low (9–11). Therefore, tools to improve the therapeutic effect of existing treatments on patients with MT is an urgent problem to be solved and a hot research topic at the moment.

Radiotherapy is one of the three most-common MT treatment modalities, along with surgery and chemotherapy. It uses ionizing radiation to kill tumor cells and shrink tumors (12, 13). Versus surgery and chemotherapy, radiotherapy uses colorless, odorless, invisible, and non-invasive radiation to kill tumor cells. It is widely used in the radical curative treatment or palliative treatment of primary MT and metastatic tumors. Approximately 70% of patients with malignancy need radiation therapy at various stages of their treatment, of which 70% are radical radiotherapy. Radiotherapy accounts for about 40% of cured malignancies (14). The goal of radiotherapy is to maximize the radiation dose to the lesion (target) area for a long time and kill tumor cells while preventing or protecting surrounding normal tissues or organs from unnecessary radiation exposure, thus providing the required special protection to some vital organs such as the brainstem, spinal cord, kidney, gonads, *etc.* (15).

Traditional radiotherapy techniques, such as Co-60 teletherapy with poor precision and limited radiotherapy effectiveness, only achieve the primary stage of the radical curative treatment of the tumor, and also cause temporary or permanent damage to normal tissues and organs (16, 17). Interventional medicine has progressed particularly rapidly and led to the development and use of interventional radiotherapy techniques, in which radiopharmaceuticals are directly injected into the lesions through intubation and injection to enrich the concentration of the drugs in the lesions. This enables precise and targeted delivery of radiopharmaceuticals and overcomes the deficiency of traditional Co-60 teletherapy (18–20). However, there are still a series of problems to be solved in using radiopharmaceuticals in interventional radiotherapies, such as the selection of radiopharmaceuticals and their carriers, the uniform distribution and long-term fixation. In addition, interventional internal radiation therapy uses padding and manual implantation of radioactive drugs by doctors with their bare hands. This can prevent accurate calculations and evaluation of the injection pressure. Surgeons are thus exposed to radiation hazards. There is also an increase in patients with radioactive leaks and absorbed dose by non-target organs when the implantation operation time is too long and the injection pressure is too high. In addition, most implantation of radiopharmaceuticals is performed under computed tomography (CT) guidance. Although CT offers high resolution, it has problems such as being unable to be used in real-time dynamic navigation, with repeat punctures or offer precise delivery.

**Figure 1:**
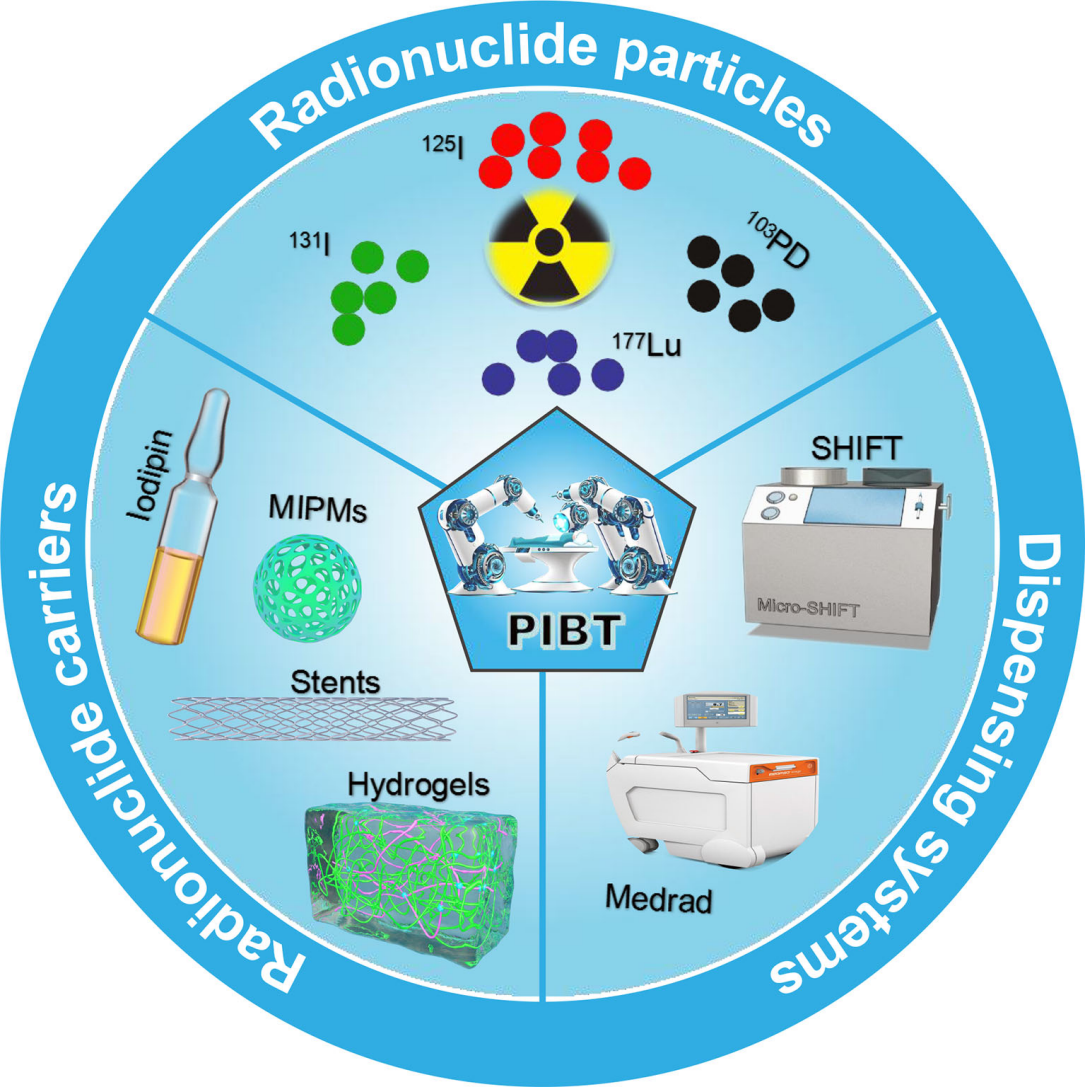
Schematic showing radionuclide particles, radionuclide carriers, and dispensing systems commonly used in interventional brachytherapy.

Precision interventional brachytherapy (PIBT) is a gradually developed radiotherapy technique that combines radiotherapy medicine with computer network technology, physics, *etc.* to address the limitations of conventional internal radiotherapy (21). For example, the most advanced radiotherapy equipment in vascular interventional robotic surgery is accurate to millimeters with very low side effects. This increases the accuracy of radiotherapy and thus application in clinical practice. Interventional doctors use catheters, guide wires, and other interventional devices to eliminate the heavy burden of lead protective aprons and reduce radiation exposure. Robot-assisted percutaneous coronary intervention (PCI) surgery can reduce radiation exposure by 97% (22). In addition, dispensing and fixation technologies for radiopharmaceuticals and drug carriers and precise delivery systems are active research topics. This paper reviews the main radiopharmaceuticals (^131^I, ^125^I, ^177^Lu, etc.), drug carriers (Lipiodol, Microspheres, Hydrogels, etc.), dispensing and fixation technologies (SHIFT, Medrad, etc.), and interventional robotic precision delivery systems used in malignant tumor PIBT (**Figure 1**). It also discusses the current needs of the field and future development prospects.

## Commonly Used Radionuclides

As early as 1901, Pierre and Marie Curie used small radium tubes for the first time to treat malignant tumors marking the birth of endoradiotherapy technology (23, 24). In 1970, Felix Mick developed a low-energy ^125^I particle source containing iodine particles encapsulated in capsules and placed in a titanium tube. The ^125^I was subsequently used for endoradiotherapy of prostate, liver, and lung cancer; its efficacy was clinically proven and widely recognized over the following decades (25–27). Versus conventional external radiotherapy, permanent ^125^I seed implantation has its unique advantages. The first is that the release of x-rays, γ-rays, and other types of radiation is from the inside of the tumor tissue, causing DNA damage to tumor cells (28). As a result, the irradiation route does not need to pass through normal tissues to reach the target area. The dose distribution follows the inverse square law with an increase as distance decreases. Thus, the surrounding normal tissues are well protected, and the incidence of complications is low. Second, the local dose is high. The intensity of the implanted radioactive source is small, and the effective irradiation radius is short; thus, a higher radiation dose can be applied to the tumor target area (29).

The commonly used nuclides in nuclear medicine mainly include ^125^I, ^103^Pd, ^169^Yb, ^198^Au, ^131^Cs, ^137^Cs, ^192^Ir, ^60^Co, *etc.* (30). New nuclides such as ^241^Am, ^152^Cf, ^26^Ra, and ^145^Sm have recently attracted considerable attention. They have been used in clinical practice, but the most commonly used nuclides are ^125^I and ^103^PD, which have become essential to traditional external radiotherapy (31). The most common models of brachytherapy source models seeds are Pharma Seed BT-125-1 or BT-125-2 (Syncor Pharmaceuticals Inc, Golden, CO, USA), ADVANTAGE™Pd-103 IAPd-103A (IsoAid LLC, Port Richey, FL, USA), Prospera I-125-Med363 (North American Scientific, Inc., Chatsworth, CA, USA), Best^®^ I-125 (Best Medical International, Inc., Springfield, VA, USA), and Type 6711 ^125^I particles (HTA Co., Ltd., Beijing, China). The diameter of ^125^I particles is 0.8 mm, and the length is 4.5 mm; the wall thickness of the enveloping titanium tube is 0.05 m (the source core is φ 0.5mm×3.0mm to adsorb ^125^I silver rod, which is suitable for killing tumor cells with slow growth). Although radionuclide particle therapy for tumors has high safety and achieves sound therapeutic effects, there are still some constraints, such as selecting radionuclide particles for the precise treatment of tumors with different proliferation rates to obtain the maximum killing effect. Second, there are complications and adverse reactions after particle implantation. Finally, there is a need to study further the efficacy evaluation methods of particle implantation combined with external radiotherapy.

## Carriers of Radionuclides for Interventional Brachytherapy

### Iodized Oil

Lipiodol is the most commonly used carrier for radionuclide drugs because it is easy to inject and selectively deposited. For example, ^131^I-labeled lipiodol has been proven to be clinically effective and is commercially available (32). However, ^131^I suffers from high-energy gamma photon emission (364 keV, 81%) (33), and the radioactivity yield of ^131^I-labeled lipiodol is also poor. Due to its suitable decay properties (T½ =6.73 days, Eβ (Max)=0.49 MeV, Eγ=208 KeV [11%]), the half-life of ^177^Lu is comparable to that of ^131^I without significant decay loss. The relatively low abundance of low-energy gamma photons can be used for simultaneous scintillation imaging and dosimetry studies without a significant additional dose burden to the patient. Thus, it is a feasible substitute of ^131^I in lipiodol for liver cancer radiotherapy (34, 35). However, water-soluble nuclide particles are difficult to disperse stably in lipiodol for a long time. For instance, Suresh et al. treated a rat orthotopic liver tumor model with ^177^Lu-labeled lipophilic 8-hydroxy-quinoline mixed with lipiodol by a traditional method. They found that it was prone to radioactive leakage and deposited in bone tissue (36). This limited the clinical application of lipiodol with ^177^Lu-labeled. Therefore, it is an important direction and hot topic for future research to develop efficient, simple, and stable lipiodol/nuclide preparations and obtain stable and long-term interventional radiotherapy.

### Microspheres

Recently, some progress has been made in developing methods for the preparation of interventional radioactive microsphere embolization materials (37). For example, Arranja et al. (38) dispersed solid acetylacetone holmium microspheres (HO2 (Acac) 3-MS) in NaH_2_PO_4_ or NaOH solutions and incubated at room temperature for 2 h to obtain two new inorganic microspheres. They then exchanged them with phosphate or hydroxyl ions through acetylacetone to obtain Ho(OH) 3-MS and Ho(OH) 4-MS. After preparing HopO4-MS and Ho(OH) 3MS, the stable isotope ^166^Ho was partially converted into radioactive ^166^Ho by neutron activation, and high activity radioactive microspheres were obtained. Zielhuis et al. (39) used elemental holmium combined with the carboxylic acid group of alginate polymer through electrostatic action to obtain alginate microspheres loaded with holmium. Finally, ^166^Ho was added into calcium-hardened alginate microspheres to obtain microspheres with high radiochemical stability (94% after 48 h incubation in human serum). Ma et al. (40) performed ^131^I labeling using gelatin microspheres as carriers, and through a study in a New Zealand rabbit liver model, found that the nuclides were aggregated in the liver in the form of microspheres after ^131^I-GMSS administration. In addition, radioactivity was detected 48 days after injection of ^131^IGMS, and the microspheres were degraded to different extents 24, 32, and 48 days after the injection of ^131^I-GMSS. Although these microspheres offer high activity and degradability, they are primarily limited to basic research at the animal level, and few radioactive microspheres can be applied in human clinical practice.

The most commonly used clinical radiation microspheres are radioactive ^90^Y microspheres. They can be injected into tumor lesions through digital subtraction angiography (DSA) super-selection, and the β-rays emitted by them can be used to kill tumors and perform endoradiotherapy (41–43). Theraspheres and SiR-spheres are available in the market. They are safe and efficacious for the treatment of TM. Several side effects are associated with trans-arterial procedures (44, 45) including dissociation of cargo and formation of ectopic embolism. Furthermore, since ^90^Y only emits beta rays and cannot be detected by single-photon emission computed tomography/positron emission tomography (SPECT/PET) imaging, it is difficult to obtain the drug distribution behavior *in vivo* by imaging techniques. There is a blind spot of the correlation between therapeutic effect and nuclide quantification. Thus, it is challenging to make unified clinical recommendations. ^90^Y microsphere treatment is economically expensive, and thus widespread use of this treatment is quite limited.

### Others

Other radiopharmaceutical carriers mainly include scaffolds, hydrogels, *etc.*, such as the intensity-modulated radiation-acrylic repositioning stent for the treatment of head and neck cancer reported by Lee Vsk et al. Retrospective cohort studies of patients with maxillary sinus, nasal, or oral cancer have revealed that acrylic repositioning stents do not alter radiotherapy outcomes and are highly stable (46). Zhu et al. (47) developed a biliary stent loaded with ^125^I radioactive particles, and used a comparative clinical study of 23 patients to show that ^125^I seeds in the biliary stent not only improved the patency of the patients’ biliary tract, but also prolonged the patients’ survival time.

Hydrogels have become a hot research topic in recent years due to their excellent biocompatibility, biodegradability, and outstanding clinical application value (48, 49). Hydrogel carriers have also played an essential role in the study of cancer brachytherapy. For instance, Schaal et al. (50) used radionuclide ^131^I to label a thermal micelle composed of an elastin-like polypeptide (ELP) to form an *in situ* hydrogel brachytherapy of prostate cancer. The study was performed on a human PC-3M-Luc-C6 prostate tumor model and human BXPC3-Luc2 pancreatic tumor model and found that the ELP pool retained 52% and more than 70% of radioactivity for 60 days in prostate and pancreatic tumors, respectively. Furthermore, after 72 h, there was no significant accumulation of radioactivity in the tissue outside the target (≦0.1%ID); the median survival time of the two groups of nude mice was significantly extended.

Puente et al. (51) used an injectable chitosan hydrogel capable of releasing a chemotherapy drug (temozolomide, TMZ) while retaining a radioactive isotope preparation (iodine, ^131^I) as the carrier of intracavity local radiotherapy and chemotherapy for the intraperitoneal therapy of brain gliomas. Some studies have shown that injectable chemical-radio-hydrogel implants can potentially improve local control and overall prognosis of invasive, poor-prognosis brain tumors. Although some studies suggest that hydrogels have many advantages, they are still limited to basic research at the animal level. These materials have not been clinically translated due to defects in drug delivery and biological behavior.

## Radiopharmaceutical Dispensing and Fixation Technology

The precise implementation of interventional brachytherapy is a significant clinical problem. The community needs to improve the dispensing efficiency and drug stability while also reducing the radiation injury to medical staff. This is an inevitable trend for nuclear medicine: Replacing manual operation with intelligent equipment (52, 53). The central dispensing systems are UG-05 (Japan), Medrad (United States), IRIS (Italy). However, such equipment has a single type of dispensing and is expensive.

**Figure 2:**
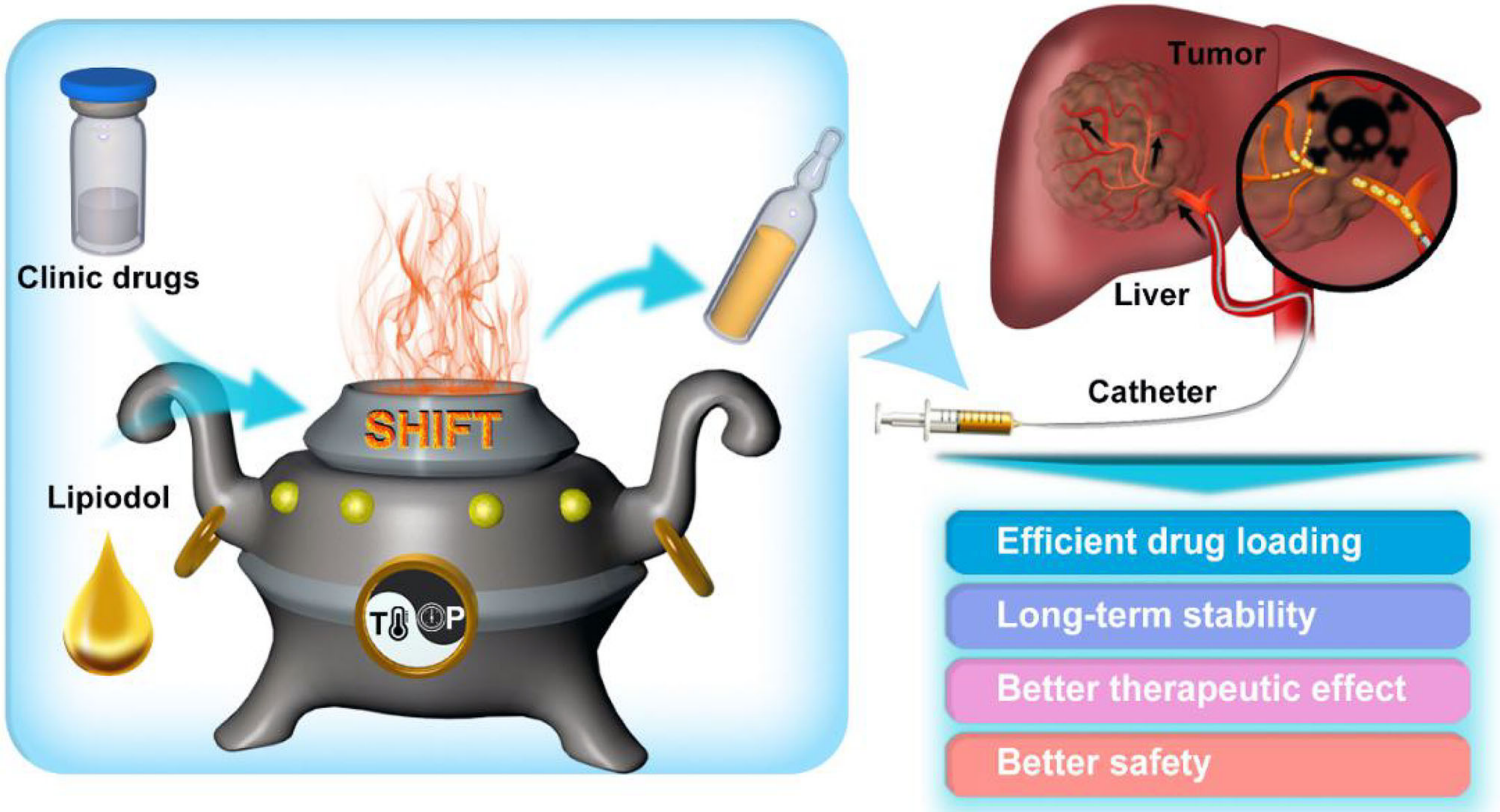
Schematic illustration of super-stable homogeneous lipiodol formulation technology (SHIFT) as a revolutionary strategy for transhepatic arterial chemotherapy and embolization (TACE). The clinic drugs and lipiodol are introduced to develop formulations with SHIFT at a controlled temperature and pressure overcoming current challenges in the hepatocellular carcinoma (HCC) treatment with TACE. (Reprinted with permission from Liu et al. (56). Copyright Elsevier B.V).

Zhang et al. successfully developed a PET molecular imaging probe microfluidic modular integrated synthesis system for the above problems. The system uses a modular microfluidic chip strategy to synthesize different positron emission tomography (PET) molecular image probes on an instrument and achieve the chemical purity and radioactive chemical purity of online controller drugs (54). The precision instrument equipment not only dramatically expands individualization and increases the accuracy of medical PET clinical applications, but also plays a vital role in the research and development of related nuclear drugs and radiation protection applications. However, this equipment is not suitable for stable mixing of radiopharmaceuticals and interventional embolic agents (such as lipiodol) commonly used in the clinic. Given this, Liu ‘s team (55, 56) from Xiamen University developed a green, chemically free, super-stable homogeneous lipiodol formulation technology (SHIFT) (**Figure 2**). This technology makes the reactor reach the state of supercritical fluid by adjusting the temperature and pressure in the reactor. One can then adjust the physical parameters such as temperature and pressure to adjust the intermolecular force of the drug. This technology not only improves the solubility of drug molecules in lipiodol, but also achieves a homogeneous and stable state for several months to offer long-term fixation of radioactive drugs and interventional embolization agents.

## Precision Delivery System of Interventional Surgery Robots

Interventional radiation therapy could be completed by an intelligent operating system. In fact, the manual operation based on experience is expected to be replaced by artificial intelligence. In order to solve the fundamental problems facing interventional radiation therapy such as intelligent precise delivery, current research mainly focuses on operator design choices, forced sensing information feedback, master-slave control methods, artificial intelligence algorithms, and the application of medical image analysis. Precision interventional robotic systems mainly include vascular interventional robot systems and particle-implantation robotic systems.

### Vascular Interventional Robotic System

The Hansen Sensei^®^ robotic system for percutaneous coronary intervention and percutaneous radiofrequency ablation was launched by Hansen Medical Inc. (Mountain View, CA, USA). It facilitates the entry and exit of the electrode conduit through the contact rolling of the friction wheel. It offers a circumferential rotation of the front end of the conduit by rotating the clamping device at the end of the conduit (57). For peripheral vascular interventional (PVI), a guidewire and a catheter should be used for drug injection. To address this, Hansen Medical Inc. updated its Magellan^®^ robotic system by adding a set of friction band components in contact with each other. They use relative friction and rolling to achieve feed rotation of the guidewire. Versus the Artisan catheter, the Magellan system is an intelligent catheter that is more refined in diameter and has better angulation with tip force feedback. The major problem with this system is that the operation requires the use of a specific catheter, and the cost of a single operation is high (58).

The CorPath^®^ GRX robot launched by Corindus Vascular Robotics Inc. (Waltham, MA, USA) is currently the only robot platform globally that can be used for PCI and PVI treatment at the same time. This robotic system manipulates the guidewire to complete the rotation and twist action of the feed by rolling and rotating the holding chamber through multiple sets of friction wheels. It then performs the rotation and feeds the guidewire through a gear transmission mechanism with position movement controlled by the manipulator’s arm. However, the current CorPath^®^ system still lacks the main end control mode. It can only facilitate the remote end speed of the guidewire tube delivery through the handle (22, 59).

In 2019, the French company Robocath SAS(Rouen France) launched the R-One^®^ robotic system for remote cardiovascular interventional therapy. The system can be used for remote delivery of coronary stents in PCI procedures. The robot is designed with a hinged open-close holding pod similar to that of CorPath^®^. However, its main-end controller can only control the execution end speed and lacks force feedback design (60).

Yang et al. conducted in-depth research on artificial intelligence. Rafi-Tari found potential operation skills of interventional surgery through an artificial intelligence framework that made the operation of the surgical robot smoother and more stable. They eventually completed the surgical task in an experiment (61, 62). Chi et al. proposed using artificial intelligence to enable the interventional surgery robot to learn from the demonstration of the operation by experts to complete the operation independently or explore autonomously within the vascular model to try to accomplish the surgical goals. The experimental results showed that artificial intelligence could achieve this goal and a more accurate and smoother operation process than manual operation (63–66).

### Particle Implantation Robot System

The Elekta-Nucletron FIRST system from Elekta-Nucletron AB (Stockholm, Sweden) includes an integrated real-time particle therapy system (FIRST™) with robot-assisted needle recovery and particle pushing devices. The system includes a computer-controlled three-dimensional (3D) transrectal ultrasound system, an integrated puncture and particle delivery device, and an integrated treatment planning system. The surgical robot was certified by the US FDA and Health Canada in 2001 and by the European Community (EC or CE) in 2002 for use only in treating prostate cancer with particle implantation (67).

The MIRA-V system (68, 69) was developed at the University of Western Ontario, Canada and is an ultrasound-guided minimally invasive robot-assisted particle implantation system for the lung. The robot carries lung dose planning software upgraded from the Prostate Particle Implantation Planning System to improve the accuracy of the execution plan (70). It also has an optical camera and a 5DOF electromagnetic tracer sensor that can monitor the position of the puncture needle tip. However, the system is still in the laboratory stage and has not been reported in clinical application. Recently, a multi-organ particle implantation surgical robot (Para-Brachyrob system) was developed by the Research Center for Industrial Robots Simulation and Testing (CESTER), Technical University of Cluj-Napoca (Cluj-Napoca, Romania) for high-dose-rate brachytherapy (71). It is still in the experimental stage and has not yet received US FDA or CE approval.

## Discussion

To summarize, research on precision interventional brachytherapy of malignant tumors has led to significant advances in the types, functions, choices, and quality of radionuclides and their carriers in radiotherapy. However, most nuclides and carriers with excellent performance are still in the basic research and animal study stage. Future efforts include optimizing the performance of existing nuclides and carriers, stabilizing the nuclides in the lesion area for a long time, improving the efficiency and safety of their use, and clinical applications.

In addition, as the key to the accurate delivery of interventional radiotherapy for malignant tumors, the interventional surgical robot has initially achieved image guidance at the technical level and realized preoperative planning, puncture, and drug configuration automatically or semi-automatically. However, clinical applications, to date, have been limited: (1) Indications are narrow. Most interventional surgical robots operate on patients with specific tumors, and surgical robots involving other malignant tumors are still in the laboratory stage. (2) The image guidance system is single-mode. Existing equipment mainly uses ultrasound, computed tomography (CT), or magnetic resonance (MR) imaging to guide implantation. There is no surgical robot with a multi-mode imaging system to guide the implantation process; thus, it is impossible to map the radiopharmaceutical distribution and pressure-gated feedback in real-time accurately. (3) The robotic system is not intelligent enough. It has not achieved the master-slave robot macro/micro composite drive or an operating system with multi-channel control feedbacks such as vision, force, and touch (haptics).

## Author Contributions

PH and SG drafted the article. ER, HWC, HC, YP, BLuo, and YX devised the concept for the mini-review along with BLi, JL, JM, and GL. All authors revised and contributed to the final version of the manuscript. PH, SG, BLi, JL, JM, and GL contributed with critical revision of the manuscript throughout the process. All authors contributed to the article and approved the submitted version.

## Funding

This work was supported by the Major State Basic Research Development Program of China (2017YFA0205201), the National Natural Science Foundation of China (81925019, 81603015, 81871404, 82003147, and U1705281), the Fundamental Research Funds for the Central Universities (20720190088 and 20720200019), the Nuclear Medicine and Molecular Imaging Key Laboratory of Sichuan province open project (nos.HYX20003), and the Chengdu Gaoxin Medical Association 2020 Annual Cancer Intervention Special Research Fund (2020S02).

## Conflict of Interest

The authors declare that the research was conducted in the absence of any commercial or financial relationships that could be construed as a potential conflict of interest.

## Publisher’s Note

All claims expressed in this article are solely those of the authors and do not necessarily represent those of their affiliated organizations, or those of the publisher, the editors and the reviewers. Any product that may be evaluated in this article, or claim that may be made by its manufacturer, is not guaranteed or endorsed by the publisher.
